# Changes of Immunological Profiles in Patients with Chronic Myeloid Leukemia in the Course of Treatment

**DOI:** 10.1155/2010/137320

**Published:** 2010-12-12

**Authors:** Zuzana Humlová, Hana Klamová, Ivana Janatková, Karin Malíčková, Petra Králíková, Ivan Šterzl, Zdeněk Roth, Eva Hamšíková, Vladimír Vonka

**Affiliations:** ^1^Department of Immunology and Microbiology, 1st Medical Faculty, Charles University, and the General Teaching Hospital in Prague, Karlovo náměstí 32, 121 11 Prague 2, Czech Republic; ^2^Department of Clinical Biochemistry and Laboratory Medicine, 1st Medical Faculty, Charles University, Karlovo náměstí 32, 121 11 Prague 2, Czech Republic; ^3^Clinical Department, Institute of Hematology and Blood Transfusion, U Nemocnice 1, 128 20 Prague 2, Czech Republic; ^4^Department of Immunology, 2nd Medical Faculty, Charles University, V Úvalu 84, 150 06 Prague 5, Czech Republic; ^5^Department of Biostatistics, National Institute of Health, Šrobárova 48, 100 00 Prague 10, Czech Republic; ^6^Department of Experimental Virology, Institute of Hematology and Blood Transfusion, U Nemocnice 1, 128 20 Prague 2, Czech Republic

## Abstract

In the previous paper of ours we compared, prior to start any treatment, a number of immunological parameters in 24 chronic myeloid leukemia patients with the same number of healthy subjects matched by age and sex. We found significant differences in the levels of immunoglobulins, the C4 component of complement, the C-reactive protein, interleukin 6, the composition of lymphocyte population and the production of some cytokines by stimulated CD3+ cells. Eleven of these patients were followed longitudinally. After treatment with hydroxyurea, interferon alpha, imatinib mesylate and dasatinib, or various combinations thereof, hematological remission was achieved in all patients and complete cytogenetic remission in nine of them. There was a nearly general tendency towards normalization of the abnormalities observed in the patients at their enrollment.

## 1. Introduction

The treatment of chronic myeloid leukemia (CML) now offers several options from which to choose. Hydroxyurea (HU) was introduced in the late 1960s and for decades remained the mainstay of palliation in CML. However, HU does not induce cytogenetic remissions in a significant percentage of patients nor does it markedly change the natural history of the disease. The adverse effects include gastrointestinal problems and cutaneous defects as leg ulcers [1], hyperpigmentation of the skin and nails, a lichen planus-like eruption, lupus erythematosus, and dermatomyositis-like eruption [2]. The first observational reports on a cytoreductive effect of interferon *α* (IFN*α*) in CML patients date back to 1980s, when IFN*α* treatment was introduced at the M.D. Anderson Cancer Center, Houston, Texas [3, 4]. IFN*α* induces durable major and even complete cytogenetic remissions (CCR) persisting for months, sometimes even for years [5]. IFN*α* not only mediates antileukemic responses via induction of T-cell immunity [6, 7], but it also promotes humoral immunity against CML antigens [8]. Some parameters of innate immunity, which apparently plays a role in anticancer immunity, are also favorably influenced by IFN*α* [9, 10]. This might elucidate the efficacy of IFN*α* treatment *in vivo* by orchestrating a network of immune cells rather than by the activation of individual populations. Other mechanisms involved in modulating the course of the disease by IFN*α* are connected with its antiproliferative effect. However, long-term treatment with IFN*α* can also produce or exacerbate immune-mediated complications [11, 12], such as cutaneous vasculitis, hemolytic anemia, thyroid gland disorders, immune-mediated thrombocytopenia, nephrotoxicity, pemphigus foliaceus, rheumatoid arthritis, systemic lupus erythematosus, and even heart dysfunction based probably on immune mechanisms [11]. A revolution into therapy of CML has been brought by the introduction of the so-called targeted drugs. The first of these disease-tailored products has been imatinib mesylate (IM) which blocks the ATP-binding pocket on the BCR-ABL tyrosine-kinase and thus prevents the activation of this enzyme which plays the key role in the pathogenesis of CML [13]. IM has been reported to have induced CCR in 74% of the newly diagnosed patients and is also active in patients previously treated with INF*α* [14]. According to a recent update, a five-year survival has been achieved in nearly 90% of CML patients [15]. However, in a portion of patients, resistance to the drug develops mostly due to the mutations in the enzyme catalytic domain [16] or as a consequence of the amplification of the *bcr-abl* fusion gene [17]. To deal with the problem, a new generation of targeted drugs is being introduced and some of its representatives are already in clinical use, for example, dasatinib [18] or nilotinib [19].

Still, neither of these drugs can cure the disease most probably due to their failure to hit the quiescent cancer stem cells. When the treatment is interrupted, the disease relapses. Many oncohematologists believe that the problem of curing CML might be unriddled by supplementing the chemotherapy with immunotherapeutic approaches. A mathematical model has been constructed suggesting that immunotherapeutic intervention tailored to the clinical condition and the underlying immune status of the patient may result in the cure of CML [20].

Although the role of immune reactions in the course of CML has been demonstrated beyond reasonable doubt, the first vaccine trials reported in the past 10 years have not been particularly successful (for review see [21]). We are of the opinion that to achieve the immunization goal it will be necessary to augment our present knowledge on the immunology of CML patients and that very likely this will lead to appreciable progress in the future immunotherapeutic undertakings. 

It was the purpose of the present study to construct immunological profiles of CML patients by testing several parameters of their innate immunity early after diagnosis, that is, prior to the start of any therapy and then to follow the influence of different therapeutic regimens on these parameters and the association of their changes with the clinical condition. In a previous paper of ours [22], representing the first part of the present study, we reported the findings obtained in 24 CML patients before the start of any therapy and in the same number of matched healthy subjects. We found a number of deviations from the norm in the immune reactivity of CML patients and significant differences between the patients' and control groups. The main differences encountered in the patients were represented by increased levels of IgA (*P* < .02), the C4 component of complement (*P* < .05), C-reactive protein (CRP) (*P* < .02), and interleukin 6 (IL-6) (*P* < .0005). Furthermore, a highly significantly decreased production of interleukin-2 (IL-2) (*P* < .0001) and tumor necrosis factor *α* (TNF*α*) (*P* < .001) in stimulated CD3+ lymphocytes and a decreased phagocytosis of killed *E. coli *by polymorphonuclears (*P* < .0001) were observed. In spite of the frequency of these aberrations, no consistent pattern which might be characteristic for CML was revealed. In the subsequent follow-up we unfortunately met considerable difficulties. First of all, we lost the majority of patients (13 of 24) for various reasons. Several of them were transplanted, a few moved out of Prague or even left the country, and some simply lost their interest in participating in the study. Another complication resulted from the dramatic progress in the therapy of CML due to the introduction of a new generation of highly effective and relatively very well-tolerated new drugs (see above). From ethical reasons, it was necessary to substitute these new drugs for the older ones. It followed that most of the patients were treated with more than one drug.

## 2. Materials and Methods

### 2.1. Patients

The basic data on 11 patients enrolled at the time of diagnosis (i.e., before starting any therapy) who remained in the study throughout are shown in Table 1. The group consisted of 5 males and 6 females, their median age was 48 years, and their age range from 33 to 62 years. The follow-up lasted for 44 to 58 months. All patients were treated either with imatinib mesylate (IM) or dasatinib (DS), but not in all of them were these drugs used as the starting therapy. In six patients, administration of tyrosine-kinase inhibitors (TKI) was preceded by interferon *α* (IFN*α*) treatment and in five patients the administration of IFN*α* followed the initial treatment with hydroxyurea (HU). In two patients, HU was given prior to IM treatment. Hematological remission was achieved in all patients and complete cytogenetic remission (CCR) was achieved in nine of them in the course of the observation period. This was associated with the decrease of lymphocyte count in nearly all the patients. The only exception was patient no. 6, with leukopenia prior to the start of the therapy, in whom the remission was associated with an increase of lymphocyte count up to the norm. At the end of the observation period, thrombocyte count was below the level of 450 × 10^9^/L in all patients. In eight of the patients the therapy was not associated with any complication. Patient no. 1, who had originally been treated with HU and who had developed symptoms of immunodeficiency (neutropenia, aphthous stomatitis, repeated infections of the upper and lower respiratory tract) in the course of the observation period, underwent a supportive therapy with growth factors (Neupogen 300 *μ*G/week) and immunoglobulins (Pasteurised Human Immunoglobulin Grifols 16% 5 mL i.m. once a week for 3 weeks). Moreover, erythematous eruptions diagnosed as erythema nodosum vasculitis developed on her feet. As an additional therapy, she received routine anti-inflammatory drugs and local corticosteroids. In this patient CCR was not achieved in spite of the IM dose having been raised to 600 mg per day.

 In patients nos. 6 and 7, laboratory tests revealed autoantibodies against thyroidal peroxidase and thyreoglobulin without clinical signs of hyper- or hypothyroidism. After switching to IM or DS, the laboratory findings normalized (see the Results section).

### 2.2. Drugs Used

Patients were treated as specified in Table 1. LITALIR (HU—Hydroxycarbamidum 500 mg, Bristol-Myers Squibb, Ltd, Prague, Czech Republic), ROFERON-A (INF-*α*-Interferon *α* 2a 18 MIU/0.6 mL inj. sol., Roche, Ltd, Prague, Czech Republic), Glivec (imatinib-mesylate Glivec, 478 mg, Novartis Europharm Ltd., Horsham, West Sussex, Great Britain), and SPRYCEL (Dasatinib 100 mg, Bristol-Myers Squibb Pharma Eeig, Uxbridge Business Park, Sanderson Road, Uxbridge, UK) were used. The dosage of the drugs administered is indicated in Table 1.

### 2.3. Blood Samplings

Before sampling, written Informed Consent was obtained from all patients and the study was approved by the Ethical Committees of the institutions concerned. In addition to samples taken for routine hematological and biochemical testing, materials for immunological assays were obtained from each subject. The first samples were taken prior to the start of any therapy. Subsequent samples were usually collected before the change in therapy. The intervals varied (with one exception, patients no. 8) from 6 to 30 months. The blood samples were distributed into tubes purchased from BD Vacutainer Systems, Belliver Industrial Estate, Plymouth, UK, as follows: (i) 7 mL of blood were taken into Z tubes, coagulated, and the serum was used for humoral immunity tests; (ii) 2 mL of blood were mixed with EDTA for immunophenotypic analysis; (iii) 2 mL of the whole blood were mixed with sodium heparine for the measuring of intracellular cytokines. The materials were tested (see below) immediately after arrival at the laboratory, except the viral antibodies, CRP and IL-6 (see below) for which all sera were tested simultaneously. Portions of the materials were preserved for further tests. Sera were stored at −20°C and leukocyte suspensions were stored in liquid nitrogen for possible future tests.

### 2.4. Immunoglobulins

The levels of the total IgG, of the IgG subclasses, of IgA, and IgM were measured by nephelometry as described previously [22]. The standard laboratory referential ranges are 6.9–14.0 g/L for IgG, 4.9 –11.4 g /L for IgG1, 1.5–6.4 g/L for IgG2, 0.2–1.1 g/L for IgG3, 0.08–1.4 g/L for IgG4, 0.7–3.7 g/L for IgA, and 0.34–2.4 g/L for IgM.

### 2.5. Autoantibodies

Antibodies against thyroidal peroxidase (ATPOAb), thyreoglobulin (ATGAb), nucleus (ANAb), mitochondria (AMAb), smooth muscles (ASMAb), cytoplasm of neutrophils (ANCAb), and endomysium (AE-GAb, AE-AAb; G refers to IgG and A refers to IgA antibody, resp.) were detected by indirect immunofluorescence as described previously [22]. We have also tested the presence of antidesmosomal antibodies (ADESAb). Only the presence or absence of antibodies was monitored, not their titers.

### 2.6. Complement

The levels of the C3 and C4 components of complement were measured by nephelometry as described previously [22]. The standard laboratory referential ranges are 0.75–1.4 g/L for C3 and 0.10–0.34 g/L for C4.

### 2.7. C-Reactive Protein

The levels of the C-reactive protein (CRP) were measured by nephelometry as described previously [22]. The standard laboratory reference range is 0.00–5.0 mg/L.

### 2.8. Interleukin-6

For the determination of interleukin 6 (IL-6), the quantitative sandwich enzyme immunoassay techniques was used as described previously [22]. The standard laboratory referential range is 3.13–12.5 *μ*g/L.

### 2.9. Subpopulations of Lymphocytes

Immunophenotypic analysis of lymphocytes was performed using monoclonal antibodies directed against the following human surface antigens: CD3, CD4, CD8, CD19, CD16, and CD56 by flow cytometry as described previously [22]. The sum of cells stained with CD4, CD8, CD19, and CD16-CD56 antibodies was considered 100%. The standard laboratory referential ranges are: 59%–84% for CD3+, 25%–59% for CD4+, 19%–48% for CD8+, 6%–22% for CD19+, 6%–30% for CD16+, CD56+ cells.

### 2.10. Intracellular Cytokine Production by Stimulated CD3+ Cells

Intracellular production of interleukin-2 (IL-2) interleukin-4 (IL-4), tumor necrosis alpha (TNF*α*), and interferon gamma (INF*γ*) in CD3+ cells stimulated by the mixture of brefeldinA and phorbol-12-myristate-13-acetate was monitored by flow cytometry as described previously [22]. The figures shown in the Results section indicate the percentages of CD3+ producing the individual cytokines.

### 2.11. Antibodies against Herpesviruses and Human Papillomaviruses

Antibodies were determined against the following herpesviruses: the herpes simplex virus type 1 and type 2 (HSV 1 and 2, IgG and IgM), the varicella-zoster virus (VZV, IgG and IgM), the human cytomegalovirus (CMV, IgG and IgM) and the EB virus (EBV, IgG and IgM against the virus capsid antigen [VCA], IgG against the virus nuclear antigen [EBNA1] and against the early antigens [EA R+D]). The following commercial kits were used: ETI-HSVK-G-1/2 and ETI-HSVK-M-1/2 (DiaSorin S.p.A., Italy) for the detection of HSV 1/2 IgG and IgM antibody, respectively; ETI-CYTOK-G Plus and ETI-CYTOK-M REV Plus (DiaSorin S.p.A., Italy ) for the detection of CMV IgG and IgM antibody, respectively; VZV IgG and VZV IgM (Nova Tec, Immunodiagnostica, GmbH Germany) for the detection of VZV IgG and IgM antibody, respectively; ETI-EBV VCA-G and ETI-EBV-VCA-M-Rev (DiaSorin S.p.A., Italy) for the detection of EBV VCA IgG and IgM antibody, respectively; ETI-EBNA-G (DiaSorin S.p.A., Italy) for the detection of EBV EBNA IgG antibody; and ETI-EA-G (DiaSorin S.p.A., Italy) for the detection of EBV EA IgG antibody. In addition, antibodies to six types of human papillomaviruses (HPV), namely, types 6, 11, 16, 18, 31, and 33 were determined using ELISA using virus-like particles as antigen as described previously [23]. Sera were diluted according to corresponding manufacturer recommendation, in case of anti-HPV antibodies 1 : 25. All samples originating from the same patient were tested simultaneously on one microplate.

### 2.12. Statistical Methods

In individual patients, Spearman's correlation coefficient was used for quantifying the monotony of trend in time of the measured items. The statistical difference *P* for the deviation from zero was calculated. When analyzing the hematologic findings for the whole group of patients, the data transformed to logarithms were analyzed by covariance analysis testing for differences among patients and the common linear regression onto the time of treatment. The linear function was used as a basic component of the experienced time trend. For comparison of the relative percentage distribution of different lymphocyte populations in the first and last samples available, **χ** ^2^ test was used. For evaluation of the production of cytokines by stimulated CD3+ cells, the mean ratio of the last and first samples available was tested by One Sample Student *t*-test for difference from 1. *P*-values have not been adjusted for multiple comparisons.

## 3. Results

### 3.1. Immunoglobulins Levels

The results of measuring the immunoglobulins levels are summarized in Table 2. Nearly generally, there was a tendency to a decrease of their levels. The decrease of total IgG for the whole group was just on the brink of statistical significance (*P* = .05). In two patients (nos. 2 and 7) the IgG levels dropped below the norm. On the other hand, a marked increase of IgGs was observed in one patient (No. 6, treated gradually with IFN*α*, IM and DS) who had had the lowest level of total IgG and pathologically low levels of IgG1, IgG2, IgG3 and IgG4 in her pretreatment serum. The treatment resulted in their restoration up to the norm. It may be of interest that in this patient the hemoglobin level dropped in the course of the observation period (see Table 1). The most frequent changes were in the IgM levels. Their slight or moderate decrease was observed in 9 patients and usually it was most marked after treatment with IM. The decrease of IgM levels for the whole group was significant (*P* = .011). Changes in IgA levels were seen less frequently. Its levels dropped significantly with time in only one patient (no. 2) and increased in other three (nos. 6, 9 and 11). The first one of these was the already mentioned patient no. 6 with an increase in all subclasses of IgG. It is noteworthy that throughout the observation period the changes in this particular patient were not associated with any marked variation in the IgM level.

### 3.2. Presence of Autoantibodies

The presence of autoantibodies is shown in Table 3. Again, no consistent pattern is apparent. Autoantibodies, which had been present in the pretreatment sera of only three patients (twice against TPO and once against SM), were detected in the course of the observation period in a total of eight patients. In most of them, their appearance was transitory. In two patients (nos. 1 and 6, both of them were females), autoantibodies were detected against three antigens: in two patients (nos. 4 and 7) against two antigens and in four patients (nos. 2, 3, 9 and 10) against one antigen only. The most frequently detected autoantibodies were reactive with TPO, TG and nucleus (in all instances in three patients). In two patients antibodies reactive with SM were detected. In 1 of these (patient no. 1), they were present in the pretreatment serum sample, disappeared after starting the therapy with HU and were not detected later on when HU was gradually replaced by IFN*α* and IM. A similar phenomenon was seen in patient no. 7, in whom the initial reactivity with TPO and TG disappeared in the course of the therapy. Thus no consistent pattern was evident and, because of the multiplicity of drugs employed for individual patients, no clear dependence on the therapy used was observed. It may be of interest that the antibodies against mitochondria (AMAb) and endomysium (E-AAb and E-GAb) were never detected. 

There seems to be some, but not quite a consistent correlation with the mode of therapy and the development of autoantibodies. Autoantibodies were detected in five of six patients treated with IFN*α* and in four out of five patients treated with HU (all of them had later on been treated with IFN*α*) but in only two out of four patients exclusively treated by TKI (either IM or DS). Furthermore, in four patients autoantibodies detected after HU and IFN*α* treatment disappeared when these drugs were replaced by IM.

### 3.3. Complement C3 and C4 Components

In the pretreatment sera normal levels of C3 were observed in all but one patient (no. 11), in whom the level was below the norm. As indicated in Table 4, little variation of C3 levels was observed in the course of the observation period; still, in three patient's a transitory decrease of its level below the standard laboratory range was observed. In all instances (including patient no. 11), their levels returned to the norm by the time hematological remission was achieved. In two of three patients with the increased levels of C4 in pre-treatment sera (nos. 4, and 5), their levels dropped in the course of treatment.

### 3.4. C-Reactive Protein and IL-6

The results are also presented in Table 4. Prior to the start of the therapy, increased levels of CRP were detected in six patients. Judging by the levels detected in the last samples collected, the levels dropped to the norm in all of them. However, it is noteworthy that in two patients (nos. 3 and 8), in spite of the hematological remission having been achieved, their levels increased, and in four other patients (nos. 1, 2, 5 and 7) a transitory increase of CRP was detected in the course of the observation period. In patient no. 7 the increased level of CRP corresponded with an acute infection of the upper respiratory tract. In the other four patients (nos. 1, 3, 5 and 7) no clinical complications at the time of CRP increase were observed or reported by the patients. As concerns IL-6 levels, there was a nearly general decrease in association with the hematological remission. The decrease for the whole group was highly significant (*P* = .001). Again, as in the previous study [22], no clear correlation between IL-6 and CRP levels was apparent.

### 3.5. Lymphocyte Subpopulations

As indicated in Table 5, in spite of the drop of lymphocytes (shown in Table 1), there was little variation in the percentage of CD3+ cells in the course of the observation period, including patient no. 6, in whom lymphopenia was detected in the pre-treatment sample (see Table 1), and patient no. 10 with CD3+ lymphocyte percentage slightly below the norm. The only exception was patient no. 11, initially treated with HU who after a rather long interval without any treatment was treated with DS. In this patient a marked drop of CD3+ cells was detected. It is clear, however, that this decrease was relative, reflecting a substantial increase of NK cells. The changes in the percentages of CD4+ and CD8+ were slight or moderate in nearly all patients and no consistent pattern was evident. Still, in four of seven patients, in whose pre-treatment samples the percentage of CD8 cells was below the norm, their percentages did not reach the lower limit of the referential range. In four patients (nos. 4, 5, 6 and 10) the remission was associated with an increase in CD19+ cells. When the distribution of lymphocyte subpopulations in the first and last sample was compared, significant differences (*P* < .05 to <.001) were encountered in six patients (nos. 1, 3, 5, 6, 9 and 11). However, no consistent pattern was apparent and thus the real significance of these findings is doubtful.

### 3.6. Intracellular Production of Cytokines

The results are presented in Table 6. Two observations may be of interest. Possibly the most important one was an increase of the CD3+ cells, which produce the cytokines, in most of the patients, including those, in whom the production of these cytokines prior to the start of the therapy had been pathologically low (e.g., in the case of IL-2, patients nos. 3 and 8). Of the ten patients, which could be evaluated, the percentage of IL-2-producing cells increased in seven, IL-4-producing cells also in seven, TNF*α*-producing cells in six and INF*γ* producing cells in seven. In some of the patients the level of cytokine production remained essentially unchanged (e.g., patient no. 8, all four cytokines) and in one patient there was a drop of IL-2 and IL-4 production (patient no. 1) and in another patient the drop of INF*γ* production (patient no. 2). 

The other noteworthy observation, closely associated with the first one, was an increase in cells producing more than one cytokine after stimulation, as reflected by an increase of the percentage sum of reactive cells. The increase was expressed in terms of the Cytokine Production Index (CPI) given by the ratio between the sum of cytokine-producing cells as detected in the last sample and the sum of cytokine producing cells in the first sample available (i.e., in all but one patient before the start of therapy). A marked increase in CPI was detected in seven (nos. 3, 4, 5, 6, 7, 9 and 10) out of 10 patients which could be evaluated. The difference for the group was highly significant (*P* = .006).

### 3.7. Antibodies Against Herpesviruses and Papillomaviruses

To clarify whether treatment of the CML patients with HU, IFN*α* or TKI was associated with activation of latent and/or persistent virus infections, we tested sera from the patients for presence of antibodies against four human herpesviruses and six human papillomaviruses. The results are summarized in Tables 7 and 8. For the sake of simplicity, only the results of testing sera taken prior to the start of any treatment and at the end of the observation period are presented. It is evident that there was only very little difference between the two sets of sera. In one patient (no. 11) treated with DS, cytomegalovirus infection was reactivated as revealed by a very marked increase in IgG antibody and the appearance of IgM antibody (results not shown).

## 4. Discussion

In the preceding paper [22], we showed that CML patients before treatment differed from matched healthy subjects in a number of immunological parameters. The major aim of the present investigation was to find out, whether these aberrations persisted, decreased, or disappeared in the course of treatment, in particular whether these changes correlated with the induction of hematological and/or cytogenetic remission, whether and how they were influenced by the drugs used for treatment, and, in the long run, what was their prognostic value, if any. Our efforts were seriously hampered by two circumstances, that is, by the loss of 13 out of 24 patients originally enrolled and, for ethical reasons, by the impossibility to maintain the original treatment regimen with either HU or INF*α*. Because of the changes in the treatment modalities, the original set of patients split into two groups. The first one consisted of eight patients originally treated with HU and/or INF*α* and subsequently with TKI, while the remaining three patients treated exclusively with TKI constituted the second group.

Of these two shortcomings, the diminution of our set of patients from 24 to 11 was certainly the more important one. This reduction increased the difficulties already inherent in the group studied, that is, its inhomogeneity (age, sex, different treatments), as regards evaluation of the data obtained. Still, because in all patients hematological remission and in nearly all of them even CCR was achieved, this bringing an element of homogeneity into the study group, some conclusions can be drawn. The most important one is the nearly general association of the remission with a normalization of the aberrant immunity parameters, both humoral a cellular. One can assume that this normalization was due to an alleviation of the CML-associated processes. The continuing followup of the patients should reveal whether any relapse of the disease would be associated with deviations from the norm and with the reappearance of the same or appearance of some other aberrations. 

As regards the levels of immunoglobulins, there was a tendency towards their decrease, predominantly IgM. The gradual decrease of IgM levels was highly significant. These findings correspond with the results reported by Steegmann et al. [24], who described considerable reduction of levels of serum immunoglobulins, including IgM, in patients previously exposed to IFN*α* and then treated with IM; according to their findings, the reduction of immunoglobulins was especially marked in patients expressing a pronounced cytogenetic response. A marked selective effect of IM treatment on serum IgM level in one patient has been reported by Nagasawa and Mizutan [25]. We are unable to provide any reasonable explanation for our observation. We can only speculate that the decrease observed was due to qualitative alteration of B cells by the therapy employed. Otherwise, no consistent pattern was apparent. We only rarely detected decrease in IgA levels, which we found significantly increased in the pretreatment CML patients when compared with their matched healthy controls [22]. 

When monitoring the presence of autoantibodies, we detected them in five out of six patients treated with INF*α*. These antibodies were directed against cytoplasm of neutrophils (ANCAb), smooth muscles (ASMAb), nucleus (ANAb), thyreoglobulin (ATG), or thyroidal peroxidase (ATPO). This is not particularly surprising, since INF*α* exhibits allo- and autostimulation activity on antigen presenting cells. However, its activity *in vivo* may depend on the simultaneous regulation of network of immune cells rather than on the activation of individual populations [10]. INF*α*, but not IM, was reported to cause an increased transcription of proteinase 3 in CD14-positive monocytes, this suggesting another possible mechanism, by which IFN*α* may promote self-antigen presentation [7]. In our study the appearance of autoantibodies was in most instances of transitory character and that their disappearance quite frequently followed the replacement of INF*α* by IM. However, autoantibodies also developed in two out of five patients untreated with INF*α*. It is therefore difficult to claim, on the basis of the present results, that INF*α* was markedly more active than TKI in inducing autoantibodies. It should be added that, in our patients, the development of antibodies did not manifest itself in any clinically recognizable disease, with the possible exception of the simultaneous presence of ANCAb and vasculitis in patient no. 1. This possible association and the subsequent cure of the vasculitis in parallel with the disappearance of the antibody after IM treatment seems to be in line with a recent report [26].

As concerns the C3 and C4 components of complement there was little variation during the observation period. Again, there was a tendency to normalization. This seems to be in agreement with other findings in patients with hematological malignancies including CML [27]; however, the changes we observed were rather small and their significance is questionable. It seems clear that the follow-up of C3 and C4 components of complement was not particularly rewarding in this study. 

The results obtained when testing the CRP and IL-6 levels are of greater interest. Both tended to decrease following the therapy. The decrease was quite common in the case of IL-6, where it was highly significant. These data suggest that a reduction of IL-6 levels might be quite a reliable marker of successful therapy. Moreover, because of its biological effects, that is, inhibition of p53-induced apoptosis [28] and suppression of phosphorylation of the retinoblastoma protein [29], its drop might contribute to the favorable course of the disease. Another favorable effect of IL-6 decrease might be associated with its role in STAT-3 activation. High levels of STAT-3 can prevent dendritic cell maturation and subsequent presentation of the antigens [30]. Thus, increased levels of IL-6 might exhibit an immunosuppressive effect. In a way, the present data correspond with the earlier observations indicating that IL-6 levels are raised in parallel with the progression of the disease into blastic phase [31, 32]. An association of remission with a decrease of CRP levels was less consistent. Although CRP levels dropped in all patients with increased levels detected in their sera taken prior to the start of the therapy, in four other patients a CRP increase was observed in the course of the observation period; in two of them it was only transitory. Because of the nature of this acute phase reactant [33–35], it is rather difficult to interpret the latter findings. In one of the patient the transitional rise of CRP was associated with a respiratory disease. In the other patients some undetected microinflammation processes might have been involved. 

Possibly the most important among our findings are the changes of cell-mediated immunity parameters associated with the achievement of remission. They were represented by a markedly increased capability of stimulated CD3+ cells to produce cytokines. Since at the time of remission, the sum of percentages of CD3+ cells producing any of the cytokines tested exceeded 100 in most of the patients, it is possible to conclude that the changes observed were mainly due to an increase in cells producing more than one cytokine. Thus, a restoration of the Tcell activities associated with the suppression of the disease was observed. Similar results were reported by Reuben et al. [36]. and Guarini et al. [37]. in patients, in whom hematologic remission was achieved by INF*α* treatment. The mechanisms responsible for the present findings are not quite clear. In this respect, two previous *in vitro* studies may be of interest and provide a lead for further studies. Pawelec et al. [38]. have reported the production of the immunosuppressive IL-10 cytokine, a potent inhibitor of type 1 cytokines, by CML cells cultivated *in vitro* and have demonstrated that its neutralization by monoclonal antibody considerably enhanced the proliferation of lymphocytes in mixed lymphocyte/tumor cell cultures [38]. In another study, Kiani et al. [39]. Showed that CD4+ cells from CML patients, which had been separated from the leukemic cells, after stimulation produced type 1 cytokines in amounts comparable to those seen in normal subjects. Taken together, these two sets of data suggest that the products of CML cells may themselves be responsible for the reduced immunocompetence of the CD3+ cells. One point deserves a special comment. All ten patients, in whom the respective data were available, had been treated, at least for some time, with IM. This drug has been reported to suppress CD3+ cell activation *in vitro* [40, 41] as well as other parameters of Tcell immunity *in vivo* [42–44]. Our findings in the present study do not seem to be in agreement with those observations. One can therefore hypothesize that the alleviation of the CML-associated processes, which was induced by the successful therapy, was a more important factor than the putative immunosuppressive effect of IM. Furthermore, it has recently been reported that IM is suppressing the activation and proliferation of CD4+CD25+ regulatory cells (T_reg_ cells) which are producing strong immunosuppressive factors like IL-10, transforming growth factor *β* (TGF*β*) and granzyme B [45]. It is thus very well possible that in the patients studied the IM-induced suppression of T_reg_ contributed to the present observation. Unfortunately, we did not monitor the levels of T_reg_ cells in the present study. It should also be recalled that a significant increase of INF*γ*-producing Tcells following IM treatment has been reported by Aswald et al. [46].

Our attempts to find a reflection of these changes in the antibody titers against herpesviruses and papillomaviruses, two virus families known to be activated under immunosuppression, failed completely. As indicated in a previous paper [22], there were no significant differences in the prevalence of antibodies against these viruses between the untreated CML patients and matched normal control subjects. In the present study the antibody patterns in sera taken before the start of therapy and after achieving remission were comparable, this strongly suggesting that in the course of the observation period the activation of latent infections did not occur or could not be revealed by the antibody tests used.

In spite of inconsistency of the treatment regimens described above, some differences in the changes of the immunological parameters studied, which might have been associated with the drugs used, were recorded. Two observations are noteworthy. First, the autoantibodies were more frequently seen in patients who had been treated with HU and INF*α* than in those treated exclusively with TKI, and they tended to disappear after substituting TKI for HU and INF*α*. Still, the lack of consistency and the small number of patients tested preclude any conclusions. Second, increase of NK cells was detected more frequently in those exclusively treated with TKI than in patients who had undergone combined therapy.

## 5. Conclusions

The present results indicate that the altered parameters of innate immunity found in the CML patients prior to the start of any therapy tended to normalize in the course of therapy leading to remission. Other data suggested, but did not prove, that some of the parameters monitored might be influenced by the treatment modality used.

## Figures and Tables

**Table 1:** Patients followed.

No.	Gender	Age^1^	Treatment			Hematologic					
			Mo^2^	Drug	Dosage/d	WBC × 10^9^/L	Lympho × 10^9^/L	Hb (gr/L)	Trc × 10^9^/L	HR^3^	CCR^3^
(1)	F	55	0	dg, HU	2000 mg	302.6	4.60	89	413	19	No
			8	IFN	5 MIU	16.2	1.45	130	188		
			16	IFN	5 MIU	69.0	3.43	131	442		
			22	IM	400 mg	10.5	2.86	124	399		
			27	IM	400 mg	7.4	1.93	120	505		
			39	IM	600 mg	4.0	1.11	120	175		
			58	DS	100 mg	2.1	0.89	119	139		

(2)	F	62	0	dg, HU	2500 mg	120.0	3.54	132	226	6	19
			6	IFN	3 MIU	4.7	1.49	151	163		
			14	IFN	3 MIU	5.1	1.20	131	352		
			15	IFN	5 MIU	5.8	0.91	140	356		
			25	IM	400 mg	3.2	1.12	138	157		
			43	IM	400 mg	7.0	1.38	130	430		
			56	IM	400 mg	9.3	1.61	130	648		

(3)	M	40	0	dg, HU	3000 mg	21.9	2.07	168	327	8	26
			7	IFN	3 MIU	8.1	1.08	163	262		
			15	IFN	5 MIU	3.1	0.85	160	125		
			26	IM	400 mg	4.2	1.10	146	246		
			43	IM	400 mg	4.5	1.37	147	209		
			58	IM	400 mg	3.9	1.01	158	210		

(4)	M	60	0	dg, HU	2000 mg	198.5	5.06	110	550	6	36
			6	IFN	3 MIU	3.4	0.86	132	115		
			12	IFN	5 MIU	3.4	1.21	137	152		
			42	IM	400 mg	5.5	1.52	131	301		
			56	IM	400 mg	3.5	1.56	136	311		

(5)	M	33	0	dg, HU	2500 mg	99.7	5.91	142	169	2	40
			6	HU	2000 mg	9.9	3.56	145	151		
			9	IFN	3 MIU	47.8	3.12	137	102		
			40	IM	400 mg	4.3	1.69	142	202		
			53	IM	400 mg	5.5	2.01	140	258		
			58	IM	400 mg	6.6	1.88	136	266		

(6)	F	35	0	dg, IFN	3 MIU	107.5	0.48	117	276	2	35
			10	IFN	3 MIU	4.4	0.76	101	182		
			12	IM	400 mg	2.8	1.12	102	145		
			36	DS	100 mg	2.9	1.61	89	233		
			48	DS	100 mg	2.8	1.83	89	331		

(7)	M	54	0	dg, HU	2000 mg	16.4	4.86	136	595	11	38
			7	HU	2000 mg	4.5	5.71	137	532		
			20	HU	1000 mg	5.5	3.21	134	209		
			34	IM	400 mg	6.9	1.23	134	206		
			47	IM	400 mg	6.8	2.19	132	236		
(8)	F	43	0	dg, IM	400 mg	26.9	4.05	144	464	2	17
			2	IM	400 mg	8.3	1.66	129	228		
			9	IM	400 mg	6.0	1.76	123	185		
			17	IM	400 mg	7.5	1.26	126	207		
			31	IM	300 mg	7.6	1.73	120	239		
			45	IM	300 mg	8.3	1.45	125	315		

(9)	M	48	0	dg, IM	400 mg	439.2	6.16	81	184	7	No
			30	IM	400 mg	4.4	1.96	150	117		
			44	IM	400 mg	5.4	1.41	149	112		

(10)	F	38	0	dg, IM	400 mg	328.6	1.81	88	614	5	27
			7	IM	400 mg	3.0	1.32	121	200		
			28	IM	400 mg	3.3	1.10	94	212		
			36	IM	400 mg	4.9	0.98	103	236		

(11)	F	61	0	dg,HU	2000 mg	16.8	5.31	109	1895	4	25
			29	DS	100 mg	5.8	2.02	100	519		
			52	DS	100 mg	5.4	2.75	104	249		

^1^Age at enrollment; ^2^Mo: month; WBC: white blood cell count; Hb: hemoglobin; and Trc, thrombocyte count; HU: hydroxyurea; IFN: interferon-alpha 2a; IM: imatinib mesylate; DS: dasatinib; MIU, Millions International Units. ^3^The figure indicates month after diagnosis at which HR or CCR was first observed; “no” means that CCR was not achieved.

Notes: Patient no. 1 developed vasculitis (erythema nodosum), neutropenia which was subsequently treated with growth factors and immunoglobulins. Patients 6 and 7 had laboratory tests positive for autoimmune thyroiditis without any clinical manifestations.

Patient no. 11 decided to undergo homeopathic therapy and self-therapy with HU and was out of the evidence for some time. At the indicated interval, DS therapy was started because of pathological findings both in peripheral blood and bone marrow.

**Table 2:** Imunoglobulins.

No.	Therapy	IgG	IgG1	IgG2	IgG3	IgG4	IgA	IgM
(1)	dg, HU	10.60	6.73	4.29	0.686	0.526	2.16	1.65
	IFN	11.10	6.14	3.74	0.767	0.364	1.95	1.50
	IFN	9.30	5.97	3.22	0.530	0.250	1.93	1.56
	IM	12.30	8.90	3.99	0.709	0.119	2.74	1.91
	IM	9.40	6.82	3.34	0.575	0.157	2.17	1.61
	IM	11.30	6.14	4.21	0.687	0.193	2.92	1.54
	DS	11.40	6.70	3.81	0.907	0.253	2.73	1.29

(2)	dg, HU	10.00	6.72	3.34	0.418	0.460	2.31	0.76
	IFN	8.58	5.00	3.34	0.353	0.339	2.12	0.72
	IFN	8.53	5.64	3.00	0.358	0.397	1.68	0.45
	IFN	9.39	4.50	3.13	0.320	0.247	1.50	0.40
	IM	8.49	4.41	2.75	0.292	0.259	1.59	0.33
	IM	6.78	3.98	2.18	0.312	0.179	1.35	0.20
	IM	6.41**	3.91**	2.24**	2.92**	1.52**	1.23**	0.23**

(3)	dg, HU	10.00	6.41	3.03	0.225	0.079	1.79	1.44
	IFN	9.19	5.83	2.74	0.196	0.082	1.66	0.83
	IFN	10.80	6.33	3.28	0.239	0.058	1.74	0.87
	IM	9.08	5.71	3.23	0.190	0.091	1.66	0.38
	IM	8.72	5.04	2.68	0.248	0.083	1.73	0.54
	IM	9.41	5.18*	3.35	0.213	0.065	1.78	0.47

(4)	dg, HU	11.60	7.79	3.94	0.666	0.833	1.86	0.94
	IFN	13.00	8.44	4.05	0.686	0.303	1.62	1.18
	IFN	13.60	10.40	3.34	0.637	0.270	1.80	0.98
	IM	10.90	6.51	3.10	0.541	0.231	1.62	0.58
	IM	10.90	6.98	3.34	0.613	0.209**	1.84	0.66

(5)	dg, HU	10.40	6.29	3.04	0.248	0.133	1.59	1.76
	HU	10.20	7.40	3.03	0.158	0.066	1.98	1.20
	IFN	9.35	6.20	2.97	0.195	0.142	1.61	1.31
	IM	9.21	5.01	2.82	0.236	0.097	1.83	1.13
	IM	9.81	5.33	3.16	0.226	0.082	1.93	1.12
	IM	9.11*	6.77	2.62	0.605	0.081	1.95	1.06**

(6)	dg, IFN	8.05	5.63	1.27	0.135	0.073	1.17	1.10
	IFN	11.30	n.t.^a^	n.t.	n.t.	n.t.	1.39	1.29
	IM	12.00	n.t.	n.t.	n.t.	n.t.	1.58	1.41
	DS	17.90	12.70	1.75	0.611	0.152	2.07	0.79
	DS	13.6*	11.20	2.17**	0.416	0.188**	2.26**	1.18

(7)	dg, HU	8.92	4.60	4.01	0.388	0.610	1.22	0.95
	HU	8.79	4.45	3.95	0.337	0.797	1.30	0.76
	HU	8.76	3.81	4.11	0.266	0.702	1.34	0.51
	IM	6.08	2.89	2.72	0.262	0.507	0.99	0.32
	IM	6.63*	31.4*	2.90	0.303	0.425	0.93	0.36*

(8)	dg, IM	12.60	9.51	3.53	0.080	n.t.	1.49	0.97
	IM	11.60	8.81	2.73	0.083	0.320	1.75	0.79
	IM	12.20	7.43	2.74	0.087	0.276	1.84	0.57
	IM	12.60	7.64	3.21	0.087	0.188	1.67	0.63
	IM	12.60	9.30	3.26	0.094	0.432	1.94	0.74
	IM	11.70	7.41	2.95	0.072	0.273	1.93	0.72
(9)	dg, IM	10.60	7.58	3.41	0.523	0.697	1.31	1.11
	IM	9.43	5.36	3.41	0.480	0.790	1.47	1.11
	IM	9.53	5.90	3.95	0.479**	0.738	1.81**	1.32

(10)	dg, IM	13.50	8.46	4.93	0.231	2.180	1.04	1.61
	IM	14.20	7.63	5.63	0.211	1.970	1.28	1.60
	IM	12.10	7.49	4.49	0.262	1.200	1.04	1.39
	IM	12.20	6.63**	4.51	0.232	1.340	1.15	1.41

(11)	dg, HU	12.00	6.21	5.66	0.231	0.146	0.88	0.67
	DS	11.50	5.97	5.63	0.271	0.085	0.91	0.45
	DS	12.40	8.12	3.50**	0.677**	0.080**	1.02**	0.32**

^a^n.t.: not tested.

*P < .05, **P < .01 *P* for trend with time.

**Table 3:** Autoantibodies.

No.	Therapy	TPOAb	TGAb	ANAb	ANCAb	AMAb	ENDOAb A	ENDOAb G	SMAb	DESMAb
(1)	dg, HU	n^a^	n	n	n	n	n	n	**p** ^ b^	n
	IFN	n	n	n	**p**	n	n	n	n	n
	IFN	n	n	n	n	n	n	n	n	**p**
	IM	n	n	n	**p**	n	n	n	n	**p**
	IM	n	n	n	**p**	n	n	n	n	**p**
	IM	n	n	n	n	n	n	n	n	**p**
	DS	n	n	n	n	n	n	n	n	**p**

(2)	dg, HU	n	n	n	n	n	n	n	n	n
	IFN	n	n	n	n	n	n	n	n	n
	IFN	n	n	n	n	n	n	n	n	n
	IFN	n	n	**p**	n	n	n	n	n	n
	IM	n	n	n	n	n	n	n	n	n
	IM	n	n	n	n	n	n	n	n	n
	IM	n	n	n	n	n	n	n	n	n

(3)	dg, HU	n	n	n	n	n	n	n	n	n
	IFN	n	n	n	n	n	n	n	n	n
	IFN	n	n	n	n	n	n	n	**p**	n
	IM	n	n	n	n	n	n	n	n	n
	IM	n	n	n	n	n	n	n	**p**	n
	IM	n	n	n	n	n	n	n	n	n

(4)	dg, HU	n	n	n	n	n	n	n	n	n
	IFN	n	n	n	n	n	n	n	n	n
	IFN	n	n	n	n	n	n	n	n	**p**
	IM	**p**	n	n	n	n	n	n	n	n
	IM	n	n	n	n	n	n	n	n	n

(5)	dg, HU	n	n	n	n	n	n	n	n	n
	HU	n	n	n	n	n	n	n	n	n
	IFN	n	n	n	n	n	n	n	n	n
	IM	n	n	n	n	n	n	n	n	n
	IM	n	n	n	n	n	n	n	n	n
	IM	n	n	n	n	n	n	n	n	n

(6)	dg, IFN	**p**	n	n	n	n	n	n	n	n
	IFN	**p**	**p**	n	n	n	n	n	n	n
	IM	**p**	n	**p**	n	n	n	n	n	n
	DS	**p**	n	n	n	n	n	n	n	n
	DS	**p**	n	n	n	n	n	n	n	n

(7)	dg, HU	**p**	**p**	n	n	n	n	n	n	n
	HU	**p**	**p**	n	n	n	n	n	n	n
	HU	n.t.^c^	n.t.	n	n	n	n	n	n	n
	IM	**p**	**n**	n	n	n	n	n	n	n
	IM	n	n	n	n	n	n	n	n	n

(8)	dg, IM	n	n	n	n	n	n	n	n	n
	IM	n	n	n	n	n	n	n	n	n
	IM	n	n	n	n	n	n	n	n	n
	IM	n	n	n	n	n	n	n	n	n
	IM	n.t.	n.t.	n	n	n	n	n	n	n
	IM	n	n	n	n	n	n	n	n	n
(9)	dg, IM	n	n	n	n	n	n	n	n	n
	IM	n	n	n	n	n	n	n	n	n
	IM	n	n	n	**p**	n	n	n	n	n

(10)	dg, IM	n	n	n	n	n	n	n	n	n
	IM	n	n	n	n	n	n	n	n	n
	IM	n	n	n	n	n	n	n	n	n
	IM	n	n	**p**	n	n	n	n	n	n

(11)	dg, HU	n	n	n	n	n	n	n	n	n
	DS	n	n	n	n	n	n	n	n	n
	DS	n	n	n	n	n	n	n	n	n

TPOAb: antibodies against thyroidal peroxidase; TGAb: antibodies against thyreoglobulin; ANAb: antinuclear antibodies; ANCAb: antibodies against the cytoplasm of neutrophils; AMAb: antimitochondrial antibodies; ENDOAb A, ENDOAb G: antibodies of the IgA and IgG classes against endomysium; SMAB: antibodies against smooth muscless; DESMAb: antidesmosomal antibodies.

^a^Negative for the respective antibodies.

^b^Positive for the respective antibody.

^c^n.t.:not tested.

**Table 4:** Complement components, CRP and IL-6.

No.	Therapy	C3	C4	CRP	IL-6
(1)	dg, HU	0.94	0.40	5.2	5.10
	IFN	0.67	0.37	3.0	2.53
	IFN	0.83	0.42	24.4	5.42
	IM	0.71	0.30	3.5	1.83
	IM	0.53	0.21	3.5	1.53
	IM	0.72	0.30	3.3	1.67
	DS	1.26	0.52	3.2	2.72

(2)	dg, HU	1.24	0.30	12.8	9.54
	IFN	1.22	0.28	3.0	3.47
	IFN	1.22	0.28	3.5	3.87
	IFN	1.15	0.25	3.5	4.92
	IM	1.19	0.26	3.2	3.21
	IM	1.27	0.27	5.4	2.45
	IM	1.20	0.24*	3.2	2.82*

(3)	dg, HU	1.19	0.26	3.0	3.9
	IFN	1.14	0.26	3.0	1.8
	IFN	0.94	0.24	3.5	1.5
	IM	1.15	0.27	3.3	2.16
	IM	1.20	0.26	3.2	1.14
	IM	1.40	0.30	32.9	3.03

(4)	dg, HU	1.35	0.38	11.6	4.40
	IFN	0.90	0.21	3.0	1.54
	IFN	1.11	0.23	3.5	1.97
	IM	1.20	0.27	3.2	1.45
	IM	1.06	0.25	3.2	1.50

(5)	dg, HU	1.03	1.23	3.0	3.30
	HU	0.97	0.21	3.5	3.67
	IFN	0.80	0.20	3.5	2.16
	IM	0.91	0.19	3.2	2.49
	IM	1.14	0.23	8.8	2.30
	IM	1.00	0.25	3.4	1.99

(6)	dg, IFN	0.98	0.21	3.0	2.80
	IFN	0.69	0.19	n.t.^a^	1.82
	IM	0.78	0.17	3.5	1.87
	DS	1.31	0.30	3.2	2.31
	DS	1.14	0.24	3.2	0.00

(7)	dg, HU	0.91	0.16	3.5	5.08
	HU	0.68	0.15	3.5	4.24
	HU	0.91	0.18	3.3	5.29
	IM	0.91	0.19	21.3	1.82
	IM	0.98	0.17	3.2	3.00

(8)	dg, IM	0.93	0.19	3.5	3.80
	IM	0.94	0.19	3.8	2.63
	IM	0.89	0.17	3.2	2.31
	IM	0.89	0.18	3.3	1.53
	IM	1.07	0.26	9.4	0.27
	IM	1.11	0.33	6.1	1.70*
(9)	dg, IM	0.86	0.29	23.0	14.20
	IM	0.91	0.24	3.2	1.21
	IM	1.05**	0.27	3.2	1.00*

(10)	dg, IM	0.79	0.30	5.9	16.71
	IM	0.86	0.24	3.3	4.97
	IM	0.81	0.17	3.2	n.t.
	IM	0.76	0.17	3.2	1.00**

(11)	dg, HU	0.65	0.36	6.8	5.20
	DS	0.59	0.25	3.2	2.23
	DS	0.80	0.35	3.4	3.50

^a^n.t.:not tested.

*P < .05, **P < .01 *P* for trend with time.

**Table 5:** Lymphocytes subpopulations.

No.	Therapy	CD3^a^	CD4	CD8	CD19	CD3-CD16,56+
(1)	dg, HU	72	54	18	14	14
	IFN	74	53	21	4	13
	IFN	84	52	32	10	6
	IM	76	48	28	11	12
	IM	76	49	24	10	12
	IM	73	47	26	13	10
	DS	79	51	27	5	14

(2)	dg, HU	86	61	25	5	7
	IFN	84	52	32	3	13
	IFN	75	50	25	4	21
	IFN	82	70	12	8	9
	IM	69	54	15	11	20
	IM	78	61	15	7	14
	IM	80	62	15	7	13

(3)	dg, HU	76	39	37	12	12
	IFN	79	42	36	8	13
	IFN	73	44	29	13	13
	IM	69	42	27	17	13
	IM	72	48	24	15	13
	IM	63	44	19	12	25

(4)	dg, HU	74	60	14	6	19
	IFN	86	59	27	6	8
	IFN	68	39	28	10	21
	IM	75	52	23	13	12
	IM	78	53	25	12	10

(5)	dg, HU	62	42	18	6	32
	HU	66	43	23	20	14
	IFN	62	40	22	24	14
	IM	65	42	23	15	20
	IM	66	41	20	13	20
	IM	67	43	24	15	17

(6)	dg, IFN	83	40	43	10	6
	IFN	77	53	23	11	11
	IM	76	52	23	11	12
	DS	76	31	43	11	12
	DS	65	29	34	23	12

(7)	dg, HU	75	51	24	10	15
	HU	81	54	27	7	12
	HU	74	48	21	11	14
	IM	73	52	20	12	14
	IM	73	53	19	10	16

(8)	dg, IM	76	54	22	9	14
	IM	72	55	17	13	16
	IM	76	54	22	8	15
	IM	77	55	19	10	11
	IM	74	54	17	9	17
	IM	74	57	17	12	14
(9)	dg, IM	85	29	53	3	12
	IM	76	34	38	7	15
	IM	74	37	34	9	15

(10)	dg, IM	67	52	14	14	16
	IM	61	40	21	20	18
	IM	55	37	16	18	25
	IM	54	37	14	23	23

(11)	dg,HU	67	57	9	20	12
	DS	59	49	9	16	23
	DS	39	32	7	10	50

^a^The figures indicate the percentages of cells positive for the respective surface CDs.

**Table 6:** Intracellular cytokines production in activated CD3+ lymphocytes.

No.	Therapy	IL-2	TNFalfa	INF-*γ*	IL-4	CPI
(1)	dg, HU	56	44	12	4	
	IFN	42	49	18	3	
	IFN	31	35	23	5	
	IM	53	60	30	2	
	IM	61	48	36	3	
	IM	n.t.^a^	n.t.	n.t.	n.t.	
	DS	32	60	23*	1	1.000

(2)	dg, HU	31	53	62	2	
	IFN	10	23	29	5	
	IFN	69	66	30	4	
	IFN	76	65	32	5	
	IM	67	73	42	6	
	IM	n.t.	n.t.	n.t.	n.t.	
	IM	55	45	35	20*	1.047

(3)	dg, HU	1	54	41	6	
	IFN	24	46	36	3	
	IFN	48	66	50	3	
	IM	23	51	25	1	
	IM	n.t.	n.t.	n.t.	n.t.	
	IM	49	57	51	20	1.735

(4)	dg, HU	17	20	25	6	
	IFN	31	46	29	8	
	IFN	38	78	38	6	
	IM	n.t.	n.t.	n.t.	n.t.	
	IM	56**	65	44**	11	2.588

(5)	dg, HU	22	21	14	4	
	HU	50	55	25	4	
	IFN	39	50	30	2	
	IM	37	49	24	8	
	IM	52	58	30	2	
	IM	38	41	30	5	1.869

(6)	dg, IFN	11	17	22	2	
	IFN	36	51	32	3	
	IM	48	55	35	5	
	DS	20	59	45	3	
	DS	18	44	37*	19	2.269

(7)	dg, HU	51	46	34	3	
	HU	52	42	30	2	
	HU	52	68	55	5	
	IM	n.t.	n.t.	n.t.	n.t.	
	IM	52	53	34	25	1.223

(8)	dg, IM	42	44	23	2	
	IM	38	44	19	2	
	IM	16	25	13	5	
	IM	n.t.	n.t.	n.t.	n.t.	
	IM	n.t.	n.t.	n.t.	n.t.	
	IM	41	37	23	3	0.937

(9)	dg, IM	2	34	45	1	
	IM	n.t.	n.t.	n.t.	n.t.	
	IM	31	74	57	13	2.134
(10)	dg, IM	n.t.	n.t.	n.t.	n.t.	
	IM	27	32	14	1	
	IM	38	54	24	3	
	IM	50**	51	30**	6**	1.827

(11)	dg, HU	n.t.	n.t.	n.t.	n.t.	
	DS	59	62	23	1	
	DS	n.t.	n.t.	n.t.	n.t.	n.t.

CPI:Cytokine Production Index—a ratio between the sum of cytokine producing cells ls as detected in the last sample and the sum of cytokine producing cells in the first sample available.

^a^n.t.:not tested.

*P < .05, **P < .01. *P* for trend with time.

**Table 7:** Presence and geometric mean titres of antibodies against human herpesviruses in CML patients prior to the start of the therapy and after achieiving hematological and/or complete cytogenetic remission.

antibody presence											
Sample	No.	HSV1/2		CMV		VZV		EBV			
		IgG	IgM	IgG	IgM	IgG	IgM	VCAIgG	VCAIgM	EAIgG	EBNA IgM

First	11	11	2	8	0	11	2	11	2	3	10
Last	11	11	0	8	1	11	2	11	2	3	10

GMT											

First	11	8.12	0.65	1.83	0.47	2.83	0.74	7.41	0.33	0.57	4.28
Last	11	8.33	0.57	2.01	0.47	2.61	0.54	8.20	0.34	0.65	4.79

**Table 8:** Presence and geometric mean titres of antibodies against human papillomaviruses in CML patients prior to the start of the therapy and after achieving hematological and/or complete cytogenetic remission.

antibody presence							
Sample	No.	HPV6	HPV11	HPV16	HPV18	HPV31	HPV33

First	11	6	5	3	3	3	1
Last	11	5	3	3	3	2	1

GMT							

First	11	1.27	1.01	0.68	1.00	0.79	0.50
Last	11	1.08	0.88	0.65	0.75	0.61	0.38
